# The Danger of a Short Sanatorium Course

**Published:** 1914-05-16

**Authors:** 


### The Hospital

The Workers' Newspaper of Administrative Medicine and Institutional Life, Administration, National Insurance and Health.

No. 1455, Vol. LVI. SATURDAY, MAY 16, 1914. PRICE ONE PENNY.

### THE DANGER OF A SHORT SANATORIUM COURSE.

The duty of affording treatment for patients suffering from pulmonary or other tuberculous disease created under the Insurance Act is being accepted by the public health authorities concerned with varying degrees of method and alacrity. The adequacy of the arrangements made and the success of the work so far accomplished have already been frequently called in question, and the root of the matter is seen to be as much one of economics as of medicine. Prominent amongst the points already at issue is that of the duration of treatment which is being, and will be, afforded to patients selected for sanatorium treatment, whether that treatment be given to insured patients or to dependants or to the uninsured. The insured have established a claim to treatment, and the right of the uninsured to participate in treatment has been accepted. A sign of what this means is seen in a report that the Public Health Committee of the London County Council have recommended that patients who receive treatment under schemes prepared by the Council or local sanitary authorities should be charged part costs in cases where the patient's income is £160 a year or over. But similar charges, to parents and others, in the case of patients suffering from infectious diseases have not generally proved to be easily or widely recoverable; and, moreover, the proposal to institute them in itself renders the patients and their relatives only the more critical of the treatment offered or afforded. No complete solution of the economic side of a long course of treatment is therefore to be found here.

The essential characteristics of modern sanatorium treatment are that it is educational, strictly disciplinary, and of sufficient duration to afford ample observation not only of the patient's condition, but of the extent and character of the reaction to sanatorium conditions of life. Further, in cases in which the improvement in response to treatment warrants, the treatment must be of long duration in order to allow of the arrest of the disease, of local changes at its seat, and of the establishment of the highest degree of power of resistance of which the patient is susceptible.

Dietary, tuberculin treatment, etc., are subordinate factors. Sanatorium treatment must differ, essentially, from rest-cure, convalescent-home treatment, or mere change of air.

This sanatorium treatment is now to be afforded upon a scale never previously attempted. It is of vital importance, therefore, to note that in the past it has depended for its success upon the selection of cases and upon the duration of the treatment; and to have been followed in the case of patients from the poorer classes only too frequently by relapse or re-infection. At present, the demand for accommodation being great and urgent, whilst the provision for it is still inadequate, an attempt is undoubtedly (and not unnaturally) being made to pass through some of the existing sanatoria as many patients as possible, and consequently and proportionately to reduce the prospects of prolonged benefit in individual cases.

It is earnestly to be hoped that those to whom is entrusted the duty of selecting and treating sanatorium patients will not allow themselves to be pressed, by a lack of accommodation for which they are not responsible, and which should be only temporary, into reducing or annulling the benefit which their patients may derive. The selection of such patients must be made with the greatest care and circumspection, regard being paid not only to physical state (important as that state is), but also to those conditions of home surroundings and work which are also to be considered in estimating the future prospects of each patient. Wasted energies and deplorable failure cannot but result when freedom and precaution of selection are interfered with. Public education is needed in the recognition and acceptance of what is to be regarded, and must by the tuberculosis medical officer be regarded, as suitability for sanatorium treatment.

The sanatorium medical superintendent has every opportunity and facility for observing the condition and the changes under treatment of his patient. In judging as to the further continuance and duration of that treatment he must exercise an unbiassed judgment; he must have ready means of obtaining full information of all such conditions as may influence the patient's future mode of life; he should be in intimate communication with the medical officer who recommended the treatment, to whom are known the details of the patient's home environment, and who will subsequently have the patient under observation at the dispensary.

The whole scheme under which tuberculosis is being attacked aims at securing, and depends very largely for its success upon, co-operation and due correlation between its divisions. By the careful selection of patients apparently suitable for strict sanatorium *régime*, and reconsideration of their suitability after the effects of treatment have been observed, the sanatorium medical superintendent and the tuberculosis medical officer will (working in unison) be able to get full value for their patients. They must freely exercise their powers of recommending the limitation or the extension of the period of treatment in the case of each individual patient. Educational courses or courses of institutional treatment for the purpose of observation must be defined as such, and must be rigorously limited.

Successful results from this form of treatment cannot be attained by any haphazard attempt to benefit a large number of carelessly classified patients by a short sanatorium course, which is certain to be followed by a high percentage of relapse and re-infection. No good will ultimately come of the sacrifice at this stage of the interests of patients of hopeful prospect in an endeavour to clear a waiting-list.

